# Designer meat production, carcass quality, and hemato-biochemical parameters of broilers fed dietary synbiotic derived from trimmed asparagus by-products combined with probiotic supplementation

**DOI:** 10.5455/javar.2022.i621

**Published:** 2022-09-30

**Authors:** Manatsanan Nopparatmaitree, Sittichai Bunlue, Silchai Washiraomornlert, Pornpan Saenphoom, Warangkana Kitpipit, Soranot Chotnipat

**Affiliations:** 1 Faculty of Animal Science and Agricultural Technology, Silpakorn University, Phetchaburi, Thailand; 2 Akkhraratchakumari Veterinary College, Walailak University, Nakhon Si Thammarat, Thailand; 3 One Health Research Center, Walailak University, Nakhon Si Thammarat, Thailand; 4 Food Technology and Innovation Research Center of Excellence, Walailak University, Nakhon Si Thammarat, Thailand

**Keywords:** Asparagus, broiler, designer meat, probiotics, synbiotics

## Abstract

**Objective:**

This experiment investigated the effects of synbiotic supplementation produced from probiotics and prebiotics from trimmed asparagus by-products (TABP) on broiler chicken diets in order to yield designer meat production.

**Materials and Methods:**

A total of 320 one-day-old Ross 308® chicks were randomly allocated to dietary treatments with four replications each (*n* = 20). The dietary treatments were composed of a control group without supplementation and treatment groups fed with 10, 30, and 50 gm/kg of TABP supplementation in diets combined with 2 gm/kg probiotics.

**Results:**

The results showed that broilers fed dietary supplementation of TABP with 2 gm/kg had a lower level of total cholesterol and low-density lipoprotein cholesterol in the serum, which reduced the atherogenic indices of the serum, such as cardiac risk ratio and atherogenic coefficient (*p* < 0.05). In addition, the cholesterol content and the fatty acid profile of breast meat, including palmitic acid, oleic acid, saturated fatty acid, and omega 9 levels, also declined with the increasing levels of TABP inclusion (*p* < 0.05). Moreover, the supplementation of TABP in diets caused a decline in the atherogenic and thrombogenicity indices and a rise in Δ-9 desaturase (16) index and hypocholesterolemic to the hypercholesterolemic ratio of meat (*p* < 0.05).

**Conclusion:**

Synbiotic supplementation of up to 30 gm/kg TABP combined with 2 gm/kg probiotics in the chicken diet can potentially be utilized for the production of designer meat.

## Introduction

The poultry production industry is entering a new era of product development toward designer food. Around 3%–5% of the yields will be classified as nutrient-rich design chicken meat and eggs or will be value-added to differentiate them from regular chicken meat and eggs [1]. Given that poultry products, such as eggs and meat, have already gained a healthy image, several attempts have been made to modify eggs and meat by adding ingredients that are beneficial to health or by eliminating or reducing harmful components. This change resulted in the creation of functional food, and the improvement of consumers’ health and nutritional status through dietary approaches is relatively simple and cost-effective. Designer chicken meat is an important category of functional food for health-conscious customers, and it can be produced in three ways: nutritionally enhanced, value-added, and added processed [2].

*Asparagus* species, belonging to the family Liliaceae, are native medicinal plants valued for their medicinal properties. In Thailand, His Majesty King Bhumibol Adulyadej the Great has initiated the promotion of *asparagus* cultivation as part of The Hub Krapong Royal Project, which is located in the Cha-am district of Phetchaburi province. During the production process, harvested *asparagus* spears are typically trimmed to select only the parts of good quality before selling to the market, resulting in trimmed *asparagus* by-products (TABP) and lower quality *asparagus* waste, which accounts for 30% to 40% of the total harvested amount [3]. *Asparagus* has low calories, high fiber content, and several phytochemicals such as cinnamic acids, saponins, flavonoids, fructans, and vitamins. The amount of fructans (inulin and fructooligosaccharide: FOS) in the *asparagus* edible portion ranges between 0.5% and 2% (dry weight) depending on the variety and is comparable to that of canning co-products (0.2% to 1.5%). However, the *Asparagus* roots contain a high concentration of reserve fructan polysaccharide, which accounts for nearly a quarter of the fresh weight [4,5]. Inulin and FOS are nondigestible feed ingredients that benefit birds by selectively changing the composition of the lower-gut microbiota and nutrient metabolism [6]. When consumed by animals, prebiotics help to stimulate fermentation in the hindgut [7], encourage the growth of beneficial microorganisms, and inhibit pathogens in the lower gut [8]. Several studies looked at the effects of prebiotics on digestibility and health benefits [9,10], which improved the performance of broiler chickens.

Probiotics are defined as live microorganisms that are beneficial for the health of broiler chicken guts. This includes *Saccharomyces* sp., *Bacillus* sp., *Lactobacillus* sp., *Bifidobacterium*, and others [11]. Previous studies done by Khalifa et al. [12] and Pourakbari et al. [13] recommend 10–20 gm/kg of probiotics in broiler feed. When consumed by the animal, probiotics provide numerous benefits, such as maintaining the balance of intestinal microflora, strengthening intestinal absorption cells, enriching nutrient digestibility [14], developing health, and improving growth performance [15]. Shulukh et al. [16] also described the effect of probiotics on animal diets for improved carcass and meat quality, lower lipid and cholesterol levels in blood serum, and increased polyunsaturated and *n*-3 fatty acid content in meat [17]. Synbiotics are created by combining and harmonizing probiotics and prebiotics [18] to encourage the growth of advantageous gut microorganisms and reduce the release of ammonia as well as improve the growth performance and survival rate [19]. Previous research has documented the effects of prebiotics and probiotics in designer chicken meat production as a healthy food for consumers. These effects include making more volatile fatty acids (VFAs) [20] and bile salt hydrolase (BSH) [21] to lower triglycerides, total cholesterol, and low-density lipoprotein (LDL) cholesterol and raising the level of high-density lipoprotein (HDL) cholesterol [22].

This study is a continuation of previous research on the effects of synbiotics derived from TABP and probiotics in the broiler chicken diet on gut ecology and feed efficiency [23]. There have been limited studies on the utilization of TABP as prebiotics in synbiotic supplementation in broiler diets. This work provides a demonstration of the synergistic relationship between TABP prebiotics and the beneficial microorganisms, resulting in effective synbiotics that work as feed additives for the improvement of nutritional values in broiler meat. The purpose of this research is to examine the effects of TABP combined with probiotic supplementation in diets on hemato-biochemical parameters, carcass characteristics, fatty acid profile, and atherogenic indices (AI) of broiler meat.

## Materials and Methods

### Ethical approval

The protocol for the experiments in this study was reviewed and approved by the Animal Care Protocol Management and Review Committee of Silpakorn University’s Faculty of Animal Science and Agricultural Technology (record no. ASAT SU0101/2562).

### Experimental design, birds, and diets

A completely randomized design was used by selecting 320 one-day-old Ross 308^®^ chicks from the hatchery for treatments.For each of the four dietary treatments, there were four replications, and 20 birds were assigned to each experimental replication. T1 *=* Control diet without supplementation (control diet) T2, T3, and T4 *=* Supplemented diets with 10, 30, and 50 gm/kg TABP combined with 2 gm/kg probiotics, respectively.

On the first day in the hatchery, all chicks received vaccinations against Marek’s disease. The vaccination of Newcastle disease and infectious bronchitis disease by eye drop method was also administered on days 7 and 14. The TABP in this experiment was collected from His Majesty the King of Thailand’s Hub Krapong Royal Project in Cha-am, Phetchaburi, Thailand. TABP was then sliced and arranged on a plastic wrap for 3 days before being baked in a 60°C oven for 3 days. Dry TABP samples were ground to a uniform 2 mm size. The chemical composition of TABPs was also examined in accordance with AOAC standards [24]. Likewise, thin layer chromatography was used to quantify the FOS based on the method of Reiffová and Nemcová [25]. According to laboratory analysis, TABP is composed of 2,175.23 kcal/kg gross energy, 86.80 % dry matter, 90.77% organic matter, 18.50% crude protein, 37.62% crude fiber, 0.61% crude fat, 0.10% total calcium, 0.66% total phosphorus, and 1.84 % FOS.

The microorganisms constituting the probiotics used in this experiment consisted of *Lactobacillus acidophilus* (1.0 × 10^10^ cfu/gm), *Lactobacillus plantarum* (1.0 × 10^10^ cfu/gm), *Saccharomyces cerevisiae* (10 × 10^9^ cfu/gm), *Bacillus subtilis* (1.0 × 10^10^ cfu/gm), *Bacillus licheniformis* (1.0 × 10^10^ cfu/gm), *Streptococcus faecium* (1.0 × 10^10^ cfu/gm), *Pediococcus pentosaceus* (1.0 × 10^9^ cfu/gm), and an added carrier to complete 1 kg. The diets were provided ad libitum during all experimental periods and created in accordance with the National Research Council’s recommendations [26]. For the first 0–21 days, the birds were fed a regular starter diet, followed by a finisher diet for the next 22–35 days (Table 1).

### Hemato-biochemical parameters and AI of serum

For sample collection, blood samples were drawn from the wing veins of 2 male and 2 female birds from each replication at 35 days of age to measure hemato-biochemical parameters. Blood was drawn and placed in two tubes, one of which was coated with EDTAK3 for hematological analysis, and another tube with no additives was used for serum biochemical testing according to the method described by Seifi et al. [27]. Serum samples prepared from the collected blood samples were centrifuged at a speed of 3,500 rpm for 10 min at a temperature of 4°C as described by Shang et al. [28]. Blood plasma was measured for hematocrit, red blood cell (RBC), white blood cell (WBC), heterophil (H), and lymphocyte (L) counts, and the H/Lratio (H/L ratio) was calculated using the Reisinger et al. [29] method. A chemistry analyzer (Advia 120, Bayer, Tarrytown, NY) was used for the determination of the following serum biochemical parameters: cholesterol and triglyceride using an enzymatic colorimetric method (CHOD-PAP method) as described by Zhao et al. [30]. The serum AI, including the atherogenic coefficient (AC) and cardiac risk ratio (CRR), were computed in the same way that Dev et al. [31] described.

**Table 1.** Ingredient composition and nutritive value of experimental diet.

Experimental diet^a^	Starter diet (0–21 days)				Finisher diet (22–35 days)			
	T1	T2	T3	T4	T1	T2	T3	T4
Ingredient composition (kg/ton)								
Maize	495.00	495.00	495.00	495.00	469.20	469.20	469.20	469.20
Soybean meal (44%CP)	365.00	363.20	359.60	356.00	309.00	307.20	303.60	300.00
TABP^b^	-	10.00	30.00	50.00	-	10.00	30.00	50.00
Probiotics^c^	-	2.00	2.00	2.00	-	2.00	2.00	2.00
Defatted rice bran	80.00	71.80	55.40	39.00	125.00	116.80	100.40	84.00
Rice bran oil	17.00	17.00	17.00	17.00	55.60	55.60	55.60	55.60
Limestone (CaCO_3_)	13.50	13.50	13.50	13.50	12.00	12.00	12.00	12.00
DCP (18%P)	21.00	21.00	21.00	21.00	19.00	19.00	19.00	19.00
Choline Chloride-L	0.30	0.30	0.30	0.30	0.30	0.30	0.30	0.30
NaCl	1.40	1.40	1.40	1.40	2.30	2.30	2.30	2.30
DL-Methionine(99%)	3.40	3.40	3.40	3.40	2.80	2.80	2.80	2.80
L-lysine (98.5%)	-	-	-	-	2.20	2.20	2.20	2.20
L-Threonine (98.5%)	1.50	1.50	1.50	1.50	0.60	0.60	0.60	0.60
Premix^d^	2.00	2.00	2.00	2.00	2.00	2.00	2.00	2.00
Total (ton)	1	1	1	1	1	1	1	1
Nutritive value from laboratory analysis (%)								
Moisture	9.65	9.69	8.98	9.33	8.87	9.24	9.13	9.51
Organic matter	91.89	92.07	92.64	93.65	93.18	92.9	93.39	92.69
Crude protein	23.56	23.91	23.71	23.57	20.49	20.36	20.41	20.33
Ether extract	5.39	5.23	5.60	5.00	5.51	5.30	5.52	5.63
Crude fiber	4.26	4.19	4.31	4.49	3.54	3.79	4.87	4.90
Gross energy (Kcal/kg)	4,079.30	4,033.60	4,012.30	4,073.90	4,089.60	4,069.90	4,148.90	4,082.00

a T1: ration without TABP, T2: ration + 10 gm/kg TABP powder; T3: ration + 30 gm/kg TABP powder, and T4: ration + 50 gm/kg TABP powder.

b TABP *=* Trimmed *asparagus* by-products.

c probiotics used in this experiment consisted of *Lactobacillus acidophilus* (1.0 × 10^10^ cfu/gm), *Lactobacillus plantarum* (1.0 × 10^10^ cfu/gm), *Pediococcus pentosaceus* (1.0 × 10^9^ cfu/gm), *Streptococcus faecium* (1.0 × 10^10^ cfu/gm), *S. cerevisiae* (10 × 10^9^ cfu/gm), *Bacillus subtilis* (1.0 × 10^10^ cfu/gm), and *Bacillus licheniformis* (1.0 × 10^10^ cfu/gm) and an added carrier to complete 1 kg.

d Each 1 kg of vitamin–mineral premix contained 20.02 MIU of retinal palmitate, 9.10 MIU of cholecalciferol, 136.50 gm of DL-3-tocopheryl acetate, 5.46 gm of phylloquinone, 5.46 gm of thiamine, 14.56 gm of riboflavin, 27.30 gm of Ca-D-pantothenate, 7.28 gm of pyridoxine, 109.20 gm of niacin, 3.64 gm of folic acid, 29.12 mg of cobalamin, 237.00 mg of D-biotin, 120 gm of manganese, 3.00 gm of selenium, 1,000 mg of zinc, 160.00 mg of copper, 400.00 mg of ferrous, and 12.50 gm of iodine.

### Carcass characteristics, meat quality, and fatty acid profile in meat

Following the completion of the experiment, four birds per experimental unit (two males and two females) were selected at random for sampling and killed by cervical dislocation for carcass and meat quality measurement.Carcasses were weighed, cut, and weighed again using the cut parts to calculate the percentage of carcass, chilled carcass, and cutting part using the method described by Faria et al. [32]. At 45 min and 24 h after slaughter, the breast meat was sampled for pH measurement using a pH meter and color measurement (at 24 h after slaughter), including lightness (L*) (0 *=* black to 100 *=* white), redness (a*) where a* denotes green (−a*) to red (+a*), and yellowness (b*) where b* denotes blue (−b*) to yellow (+b*) by a Minota 410 Chromameter. The hue angle and chroma were calculated using the L*, a*, and b*, as described by Pathare et al. [33]. Drip, boiling, thawing, and roasting losses were measured as an indication of the water holding capacity, following the procedure according to the previously established method described by Barbosa et al. [34]. The total cholesterol was analyzed by method C45,994.10 following AOAC standards [24]. The separation and analysis of individual fatty acid content were performed using gas chromatography (HP6,890; Agilent, Waldbronn, Germany) in accordance with the method described by Lepage and Roy [35]. The fatty acid content in breast meat was used to calculate the quality of fat, specifically the Δ-9 desaturase (16) index, Δ-9 desaturase (18) index, and AI according to He et al. [36]. Additionally, the Zhai et al. [37] method was used to calculate the iodine value and the ratio of saturated fatty acid to unsaturated fatty acid (SFA/USFA). In addition, the hypocholesterolemic to hypercholesterolemic ratio (h/H ratio) and thrombogenicity index (TI) were calculated utilizing the Loponte et al. [38] method.

### Statistical analyses

Data analysis was performed using the analysis of variance (ANOVA) for a completely randomized design using the method described by Steel and Torrie [39]. When the ANOVA results showed significant differences, Tukey’s honestly significant test was then applied according to the R Core Team [40]’s description of *R* program version 3.5.1. The following statistical model was utilized in this experiment: *Y*_ij_ *=* µ + *T*_i_ + *e*_ij_ where μ *=* general mean, *T*_i_ *=* effect of treatment (*i* *=* control group and TABP supplementation at 10, 30, and 50 gm/kg combined with probiotics 2 gm/kg), and *e*_ij_ *=* random error associated with *Y*_ij_ observation.

## Results

### Hemato-biochemical parameters and AI of serum

In the broiler chicken diets, TABP supplementation in conjunction with 2 gm/kg total probiotics had no impact on blood parameters (*p >* 0.05). Additionally, this experiment showed that broilers fed diets containing supplements of 10, 30, and 50 gm/kg TABP in addition to 2 gm/kg probiotics had lower serum levels of total cholesterol and LDL cholesterol than the control group (*p <* 0.05). Nonetheless, a quadratic trend (*p* *<* 0.05) in serum total cholesterol and LDL content was seen after increasing TABP supplementation with 2 gm/kg probiotics (Table 2). Furthermore, broilers fed a diet having 30 and 50 gm/kg TABP with 2 gm/kg probiotics had a lower CRR in their serum than the control group (*p <* 0.05). Table 2 demonstrates that the CRR and AC in serum displayed a quadratic trend when any of the TABP supplements were combined with 2 gm/kg probiotics (*p <* 0.05).

### Carcass characteristics, meat quality, and meat fatty acid profile

The carcass percentage, percentage of cutting, pH, meat color including L*, a*, b*, hue angle, and chroma, as well as the water holding capacity of broiler meat from all treatments of TABP supplemented with a combination of 2 gm/kg total probiotics were the same as the control group (*p >* 0.05). Broilers fed with 50 gm/kg TABP in combination with 2 gm/kg total probiotics in their diets had lower cholesterol content than the control group (*p <* 0.01). Combining TABP supplementation with 2 gm/kg probiotics in diets resulted in a quadratic reduction in cholesterol in chicken meat (quadratic, *p <* 0.01; Table 3). Furthermore, in contrast to the control group, broilers fed with diets containing 30 and 50 gm/kg TABP in addition to 2 gm/kg probiotics had lower levels of SFA, palmitic acid, palmitoleic acid, and omega-9 fatty acids in meat (*p <* 0.01). Supplementing the diets with 10, 30, and 50 gm/kg TABP in combination with 2 gm/kg probiotics resulted in a linear decrease in SFA, palmitic acid, and palmitoleic acid in meat (*p <* 0.01), whereas oleic acid and omega-9 fatty acids in meat resulted in a quadratic decrease (*p <* 0.01). Additionally, this study illustrated the impact of TABP supplementation with 2 gm/kg total probiotics on functional broiler diets, with broilers fed with diets containing 10, 30, and 50 gm/kg TABP in combination with 2 gm/kg probiotics showing a lower AI of meat than the control group (*p <* 0.05). The reduction of AI in chicken meat followed a quadratic curve pattern with the increase of TABP supplementation in broiler diets along with a total probiotic dose of 2 gm/kg (*p <* 0.05). Moreover, broilers fed with diets containing TABP supplements at the doses of 10, 30, and 50 gm/kg along with 2 gm/kg probiotics yielded meat with a higher desaturase 16 index and h/H ratio than the control group (*p <* 0.05) as well as a quadratic increase in Δ-9 desaturase (16) index and h/H ratio of meat (*p <* 0.05). However, there was no discernible difference in the Δ-9 desaturase (18) of the meat in comparison to the treatment groups (*p >* 0.05; Table 4).

**Table 2.** Effects of TABP and probiotics supplementation in diets on hemato-biochemical parameters.

Hematology	Control	Level of TABP with 0.2% probiotics supplementation in diets (gm/kg)			SEM	*p*-value	Trend analysis
		10	30	50			
Blood parameter							
WBC (10^9^/mm^3^)	15.93	46.00	29.0	21.13	3.88	0.10	NS
L (%)	54.67	54.00	59.67	44.33	2.97	0.38	NS
Heterophile (%)	35.33	37.67	31.67	50.33	3.41	0.31	NS
H/L ratio	0.66	0.71	0.54	1.36	0.13	0.20	NS
RBC (× 10^6^/mm^3^)	1.65	2.02	2.21	1.85	0.16	0.64	NS
Hemoglobin (gm/dl)	13.00	27.67	31.00	25.00	2.37	0.65	NS
Hematocrit (%)	13.00	13.07	10.83	13.33	0.57	0.42	NS
Serum biochemistry (mg/dL)							
Cholesterol	178.50^a^	164.50^bc^	160.00^c^	166.33^b^	2.07	0.02	Q2
HDL cholesterol	119.00	123.67	113.67	118.00	3.73	0.82	NS
LDL cholesterol	53.00^a^	38.00^bc^	34.00^c^	42.00^b^	1.91	0.03	Q2
Triglyceride	60.33	63.76	60.67	61.00	1.75	0.90	NS
AI of serum							
CRR	1.56^a^	1.40^b^	1.41^b^	1.49^ab^	0.02	0.03	Q2
AC	0.56^a^	0.41^b^	0.41^b^	0.50^ab^	0.02	0.03	Q2

SEM* =* Standard error of mean, NS* =* Not significantly different (*p* > 0.05), Q2 *=* Quadratic.

a,b,c Mean with symbol within same row differ significantly different (*p* < 0.05).

## Discussion

This research discovered that synbiotics derived from TABP combined with probiotic supplementation did not significantly affect the resulting carcass traits or meat characteristics, consistent with previous reports by Wang and Zhou [41]; Dev et al. [31]. Supplementation with a broiler diet with prebiotics and synbiotics also had no effect on the carcass, thigh, breast percentage, or internal organs such as the liver, heart, and small intestine [42]. Furthermore, these studies’ findings were in accordance with those of Tayeri et al. [43], which reported that synbiotic supplementation had no impact on carcass percentage or abdominal fat. In addition, the combination of inulin-derived synbiotics with probiotics had no effect on the shear force, meat water holding capacity, and meat color (L* a* and b*) [44]. Even though a certain amount of cholesterol in the bloodstream is good for health, too much cholesterol can increase the risk of heart disease and the hardening of the arteries.

The findings of this experiment showed the benefits of TABP synbiotic supplementation with 2 gm/kg of probiotics in lowering LDL cholesterol and triglycerides in the serum and the amount of cholesterol in chicken breast meat. Synbiotics are a mix of probiotics and prebiotics that have been linked to lower serum cholesterol in three different hypotheses. The cholesterol-lowering mechanism of probiotics is classified as an indirect process, and it is thought to be due to the functional synergy between probiotics and prebiotics. This synergistic relationship affects BSH, which facilitates the dissociation of conjugated bile salts toward the unconjugated acidic forms (either glycol-bile acids or tauro-bile acids), resulting in increased bile excretion in the stool [45]. Thus, the increased use of cholesterol in the bile production process results in lower cholesterol levels [46]. Furthermore, dietary fiber prebiotics interferes with the bile reabsorption in the liver by elevating the bile excretion and the resulting bile synthesis by 7-hydroxylase [47]. The ability of probiotics to use cholesterol for metabolism while decreasing cholesterol absorption from the gastrointestinal tract can be explained by Dev et al. [31]. Tjandrawinata et al. [48] described bacteria that have a cholesterol uptake mechanism and can bind cholesterol to cell walls by binding to the phospholipid bilayer of probiotic cells. Growing probiotic cells can store cholesterol that is permanently attached to other cholesterol [49]. Cholesterol is eliminated by both probiotics that live during growth and dead cells. However, their ability to reduce cholesterol content is less than that of growing probiotics [45]. According to Dev et al. [31], prebiotics increases the gastrointestinal viscosity and the thickness of the intestinal mucosal layer, which inhibits cholesterol absorption and may lead to increased metabolism of cholesterol in the liver, causing a decrease in hypocholesterolemia. The concentration of short-chain fatty acids is increased due to the prebiotic effect, which limits the synthesis of cholesterol or triglycerides in the hepatic. The activity of the hydroxymethylglutaryl coenzyme A (HMG-CoA) is inhibited in both probiotic and prebiotic activity, which is related to cholesterol biosynthesis. The reduction or inhibition of HMG-CoA reductase activity is related to acetylsalicylic acid [50]. While the levels of cholesterol are low in the liver, the synthesis of cholesterol is stimulated by HMG-CoA reductase and the production of LDL receptors in the liver, which move the cholesterol molecules from the blood into the hepatic cells, thereby affecting the cholesterol levels in the bloodstream and cholesterol accumulation [51]. Acetic acid is converted to acetyl-CoA, which is involved in the hepatic cholesterol synthesis pathway [47]. Butyric acid oxidizes the mitochondrial fatty acids into acetyl-CoA products, prevents hepatic cholesterol synthesis, and serves as a source of energy for intestinal epithelial cells. Contrastingly, propionic acid may inhibit fatty acid synthesis and lower cholesterol synthesis in the liver, affecting the triacylglycerol secretion rate [48]. Mistry et al. [52] discovered an increase in the acetic and butyric acid levels in the manure of mice that were fed inulin, and the administration of propionic acid to hepatocytes *in vitro* reduced cholesterol synthesis. As a result, cholesterol levels in the blood are low [53]. The mechanism of cholesterol-to-coprostanol conversion using cholesterol reductase was described by Ooi and Liong [54], who also demonstrated how inhibition of HMG-CoA reductase is linked to the cholesterol synthesis pathway. The results of this trial were similar to previous ones in terms of lowering serum cholesterol by supplementing probiotics, prebiotics from oligosaccharides and inulin [55], and prebiotic supplementation from *Spirulina platensis* [14]. Furthermore, these findings demonstrated the effect of broiler diets containing TABP supplementation with 2 gm/kg of probiotics on reductions in total cholesterol and LDL cholesterol, as well as their ability to raise HDL cholesterol levels in the serum. The results of this trial were similar to those of previous studies indicating the potential of synbiotic additives to decrease total cholesterol, LDL cholesterol, and triglyceride levels in serum [56]. In addition, synbiotics also increase serum HDL cholesterol levels [44]. Tufan and Bolacali [57] observed that synbiotic supplementation in quail diets reduced triglyceride and total serum cholesterol levels while increasing HDL cholesterol [58]. Moreover, with the addition of synbiotics [59], several previous studies found different ways of feeding synbiotics to broilers without affecting the total or LDL cholesterol levels in the serum [60]. SFAs in the diet play an important role in lowering cholesterol levels associated with coronary artery disease, and PUFAs can help lower LDL levels in plasma and the total ratio of HDL cholesterol [61]. Several previous studies attempted to find a definitive explanation of the mechanism for regulating fatty acid composition in meat as a result of nutribiotic action in diets such as synbiotics, probiotics, and prebiotics. According to Reis et al. [62], FOS and inulin have different effects on fatty acids due to the level of expression and/or activity of enzymes on various oligosaccharide derivatives. One possible explanation is that hepatic desaturation is activated during the synthesis of oleic acid from stearic acid [63]. Individual fatty acids have diverse biological effects, several of which affect the risk of cardiovascular disease (CVD). Shramko et al. [64] discovered a link between C16:0 fatty acids, the increase in consumer blood cholesterol, and C14:0, which is more inductive than C16:0, whereas C18:0 has no effect on LDL and HDL changes. Bonos et al. [65] say that the fatty acid composition of meat can be changed by microorganisms in the gut that can convert USFAs to SFAs through hydrogenation.

**Table 3.** Effects of TABP and probiotics supplementation in diets on carcass and meat quality.

Carcass percentage and meat quality	Control	Level of TABP with 0.2% probiotics supplementation in diets (gm/kg)			SEM	*p*-value	Trend analysis
		10	30	50			
Carcass and cutting percentage (%)							
Thai carcass	84.02	86.14	84.21	82.96	0.48	0.21	NS
Dressing carcass	75.95	78.20	75.86	75.87	0.48	0.21	NS
Chill carcass	74.43	76.64	74.34	74.35	0.41	0.21	NS
Breast	25.20	25.38	24.47	25.05	0.56	0.94	NS
Fillets	4.56	5.57	4.62	4.87	0.11	0.06	NS
Wing	10.88	10.82	10.84	11.24	0.15	0.63	NS
Thigh	16.81	17.74	17.45	18.25	0.31	0.48	NS
Drum strict	11.01	11.14	11.55	11.65	0.17	0.52	NS
Head	6.41	6.18	5.99	5.76	0.22	0.75	NS
Shank	3.35	3.34	3.39	3.43	0.06	0.95	NS
Skeletal	18.91	17.53	19.56	18.54	0.68	0.76	NS
Internal organ	11.82	10.89	12.88	11.57	0.42	0.45	NS
Meat quality							
pH 0	6.21	6.24	6.18	6.16	0.18	0.72	NS
pH 24	5.92	5.87	5.89	5.90	0.21	0.64	NS
Color at 24 h after chilled storage at 4°C							
−L* (Lightness)	55.87	55.81	55.34	54.52	0.81	0.93	NS
−a* (Redness)	0.36	0.59	0.30	0.24	0.20	0.46	NS
−b* (Yellowness)	9.76	9.65	11.95	10.89	0.52	0.41	NS
−Chroma	0.001	0.004	0.001	0.001	0.00	0.05	NS
−Hue angle	1.53	1.51	1.55	1.55	0.09	0.16	NS
Water holding capacity (%)							
−Drip loss	4.55	3.74	4.08	4.77	0.25	0.48	NS
−Cooking loss	21.75	22.71	24.36	23.88	0.84	0.70	NS
−Thawing loss	10.01	7.24	8.25	8.06	0.52	0.34	NS
−Roasting loss	21.06	20.71	22.28	20.72	1.01	0.94	NS

SEM* =* Standard error of mean, NS* =* Not significantly different (*p* > 0.05).

**Table 4.** Effects of TABP and probiotics supplementation in diets on fatty acid profile in meat.

Fatty acid profile in meat and quality of fat in meat	Control	Level of TABP with 0.2% probiotics supplementation in diets (gm/kg)			SEM	*p*-value	Trend analysis
		10	30	50			
Cholesterol (gm/100 gm)	77.50^A^	76.03^AB^	74.93^AB^	70.93^B^	2.95	0.04	Q2
Fatty acid profile in meat (gm/100 gm)							
Myristic acid	0.01	0.01	0.01	0.01	0.00	0.00	NS
Palmitic acid	0.48^A^	0.37^B^	0.30^B^	0.28^B^	0.04	0.02	*L*
Palmitoleic acid	0.07^A^	0.06^AB^	0.06^AB^	0.05^B^	0.01	0.01	*L*
Stearic acid	0.14	0.14	0.13	0.13	0.01	0.11	NS
Vaccenic acid	0.03	0.03	0.03	0.03	0.00	1.00	NS
Oleic acid	0.51^A^	0.57^AB^	0.51^B^	0.53^B^	0.03	<0.01	Q2
Linoleic acid	0.23	0.22	0.22	0.19	0.02	0.06	NS
Linolenic acid	0.01	0.01	0.01	0.01	0.00	0.00	NS
Eicosenoic acid	0.01	0.01	0.01	0.01	0.00	0.00	NS
Arachidonic acid	0.01	0.01	0.01	0.01	0.00	0.00	NS
∑SFA	0.63^A^	0.52^AB^	0.42^B^	0.42^B^	0.05	<0.01	*L*
∑MUFA	0.63	0.63	0.63	0.63	0.04	0.11	NS
∑PUFA	0.26	0.24	0.25	0.21	0.02	0.06	NS
∑Omega 3	0.01	0.01	0.01	0.01	0.00	0.00	NS
∑Omega 6	0.25	0.23	0.24	0.20	0.02	0.06	NS
∑Omega 9	0.52^A^	0.49^B^	0.39^C^	0.39^C^	0.03	0.01	Q2
Quality of fat in meat							
*n*3/*n*6 ratio	0.04	0.04	0.04	0.05	0.04	0.06	NS
SFA/USFA ratio	0.04	0.04	0.04	0.04	0.07	0.61	NS
Iodine value	0.98^A^	1.01^AB^	0.96^B^	0.91^C^	0.05	<0.01	*L*
AI	0.55^A^	0.47^B^	0.38^C^	0.37^C^	0.03	0.04	Q2
Δ-9 desaturase (16) index	13.77^b^	15.08^a^	16.65^a^	15.00^a^	0.08	0.04	Q2
Δ-9 desaturase (18) index	78.19	80.66	81.91	80.09	2.29	0.49	NS
TI	1.12^a^	1.07^ab^	0.96^b^	1.01^b^	0.04	0.03	Q2
h/H ratio	1.83^b^	1.88^ab^	2.05^a^	2.05^a^	0.07	0.04	Q2

SEM* =* Standard error of mean, NS* =* Not significantly different (*p* > 0.05), Q2 *=* Quadratic.

a,b Mean with symbol within the same row differ significantly different (*p* < 0.05).

A,B,C Mean with symbol within same row differ significantly different (*p* < 0.01).

This study also demonstrated the effect of TABP supplemented with 2 gm/kg probiotics in broiler chicken diets on decreasing palmitic acid, oleic acid, SFA, and MUFA content, whereas no effect was observed on omega-3 and omega-6 fatty acids. The results were in line with a prior investigation into the results of prebiotic supplementation on linoleic acid content, increased PUFA content in meat of the breast and thigh part, and decreased arachidonic acid content in pectoralis major meat. Furthermore, the effects of probiotic supplementation on lowering oleic acid, arachidonic acid, SFAs, stearic acid proportion, CVD, MUFA, and omega-6 fatty acids in chicken meat and reducing oleic acid content in rat liver [66,67] were observed. Furthermore, this trial showed a lower CVD risk index and concluded that meat from broilers fed with a diet containing synbiotics from TAPB plus probiotic supplementation has good quality lipids and is suitable as a functional diet to lower the risk of the noncommunicable syndrome. Dev et al. [31] reported changes in cardiac protection indexes, such as CRR, AC, and AI. The increased absorption of dietary nutrition and antioxidants, which reduced postprandial lipids linked to oxidative damage, heart disease, and other vascular diseases, was the primary cause of this phenomenon. Ross et al. [68] provided experimental results on the effect of probiotics on the reduction of the AI value, which is used as an indicator of functional foods that are similar to the current findings. The results of this trial also demonstrated that synbiotics from TABP supplemented with 2 gm/kg probiotics in broiler chicken feed enhanced the metabolic disease inhibition index, both Δ-9 desaturase index and h/H ratio, which agrees with Dev et al. [69], who described that the h/H ratio increased when animals were given 0.2% prebiotics of MOS.

## Conclusion

This study also highlighted the benefits of synbiotics from TABP in combination with 2 gm/kg of probiotics in broiler diets for lowering total cholesterol, LDL cholesterol, CRR, and AC in serum and lowering total cholesterol, palmitic acid, oleic acid, SFA, and omega-9 fatty acids in chicken breast meat. Additionally, supplementing synbiotics from TABP with probiotics as functional feed for broilers can reduce AI and TI while increasing the Δ-9 desaturase (16) index and h/H ratio. The decrease in AI and TI of broiler meat is an important indicator of functional food. This study showed that supplementing diets with synbiotics of 30 gm/kg TABP combined with 2 gm/kg probiotics could potentially be utilized as a functional additive for broiler chickens to produce designer meat.

## List of Abbreviations

AC, atherogenic coefficient; AI, atherogenic; BSH, bile salt hydrolase; CRR, cardiac risk ratio; CVD, cardiovascular disease; FOS, fructooligosaccharide; H; heterophil; h/H ratio, hypocholesterolemic to hypercholesterolemic ratio; HDL; increase high-density; L*, lightness; a*, redness; b*, yellowness; HMG-CoA, hydroxymethylglutaryl coenzyme A; L; lymphocyte; LDL, low-density lipoprotein; MOS, mannoooligosaccharide; MUFA, monounsaturated fatty acid; PUFA, polyunsaturated fatty acid; RBC; red blood cell; SCVFAs, short chain volatile fatty acids; SFA, saturated fatty acid; TABP, trimmed *asparagus* by-products; TI, thrombogenicity index; USFA, unsaturated fatty acid; VFAs, volatile fatty acids;WBC; white blood cell.
